# Challenges in the Management of Perianal Hidradenitis Suppurativa in an African American Male: A Case Report

**DOI:** 10.7759/cureus.45788

**Published:** 2023-09-22

**Authors:** Natalie Russell, Ngumimi Kpughur-Tule, Emmanuel Ravichandar, Gerard Dorta Torres, Frederick Tiesenga

**Affiliations:** 1 Clinical Sciences, Saint James School of Medicine, Chicago, USA; 2 General Surgery, West Suburban Medical Center, Chicago, USA

**Keywords:** chronic disease, mental health, dapsone, acne inversa, hidradenitis suppurativa

## Abstract

Hidradenitis suppurativa (HS), more commonly known as acne inversa, occurs due to chronic inflammation of the body’s apocrine glands, most commonly affecting areas of the body where there is prolonged skin-on-skin friction. HS affects approximately 4% of the United States population, most commonly women in their second or third decade of life, especially those of African American ethnicity. HS presents as tender subcutaneous nodules which often rupture, leading to the formation of painful dermal abscesses which undergo fibrosis and lead to the formation of extensive sinus tracts, a phenomenon known as “tunneling”. HS is staged clinically using the Hurley staging system, where the stage determines what treatment modalities are used. These modalities can include medical management such as antibiotics, or biologics such as tumor necrosis factor (TNF)-alpha inhibitors like adalimumab, as well as surgical options including incision with or without drainage. Due to the similar presentation of HS with other conditions, this disease is commonly misdiagnosed, often leading to delayed treatment initiation and worse outcomes for patients. Presented is a case report of a 30-year-old African American male with perianal HS and the potential long-term complications and challenges of management of this disease.

## Introduction

Hidradenitis suppurativa (HS), also known as acne inversa, is a chronic progressive inflammatory disorder of the terminal follicular epithelium in the apocrine skin glands causing painful bumps on the skin that can often result in infection due to friction of the skin allowing them to open [1]. The most commonly affected locations of this disease are those that have high levels of prolonged skin-to-skin contact, such as the underarms, groin, between the buttocks, under the breasts, within stomach folds, and in the nape of the neck [1]. Reports estimate that in the United States, only one out of every 100 people are being diagnosed with the disease, making it uncommon, possibly due to it being underreported [1]. The majority of people diagnosed with HS have a mean age at onset of 21.8 years of age; however, there have been reports of initial presentation into the fourth decade of life as well. In addition, the median age at onset for females was earlier than for males, with females averaging onset at 19 years of age and males at 23 years of age [2]. It has also been reported that African Americans and biracial individuals are three times more likely to develop this disease compared to Caucasians and those with a low socioeconomic status due to limited access to healthcare ultimately leading to delayed diagnosis and treatment initiation [1]. Diagnosis of HS can be complicated, as it can be mistaken for other conditions such as ingrown hairs or infections which can prolong diagnosis. Taking a proper patient history and swabbing any drainage for analysis can expedite the diagnosis [3]. There is currently no cure for HS; however, lifestyle modifications and medication therapy can reduce the number of flare-ups experienced by patients. Reducing risk factors such as losing weight, consuming low glycemic foods, quitting smoking, maintaining proper hygiene, and wearing loose clothing are all known ways to help reduce the occurrence of outbreaks [4]. The use of warm compresses and pain medications such as nonsteroidal anti-inflammatory drugs (NSAIDs) and corticosteroids has also been documented as treatments, but the effectiveness of treatment widely varies among patients as no single treatment has proven to be effective, making this disease challenging to treat [4]. In some cases, surgery may be needed for disease management [3]. Currently, the only United States Food and Drug Administration (FDA)-approved medication for the treatment of HS is Humira (adalimumab), a tumor necrosis factor (TNF)-alpha inhibitor used in the treatment of moderate to severe disease in people ages 12 and older [4]. The use of dapsone has recently been strongly considered due to its effects as both an anti-inflammatory and an antimicrobial; however, more research is needed to create and approve additional medications to be used as treatment [5]. Complications of this disease are vast and can include both local and systemic infections, abscesses, and long-term psychological effects such as anxiety, depression, and social isolation due to the chronicity of this condition.

Presented in the following is a case report of a 30-year-old African American man with a past medical history of HS ultimately leading to the formation of a left perianal abscess requiring surgical debridement and multiple wound care visits thereafter. The use of dapsone as a treatment option was considered for his condition, but ultimately not used due to low normal glucose-6-phosphate dehydrogenase (G6PD) levels. The patient was in turn started on Infliximab, also known as Remicade, a TNF-alpha inhibitor medication.

## Case presentation

**Figure 1:**
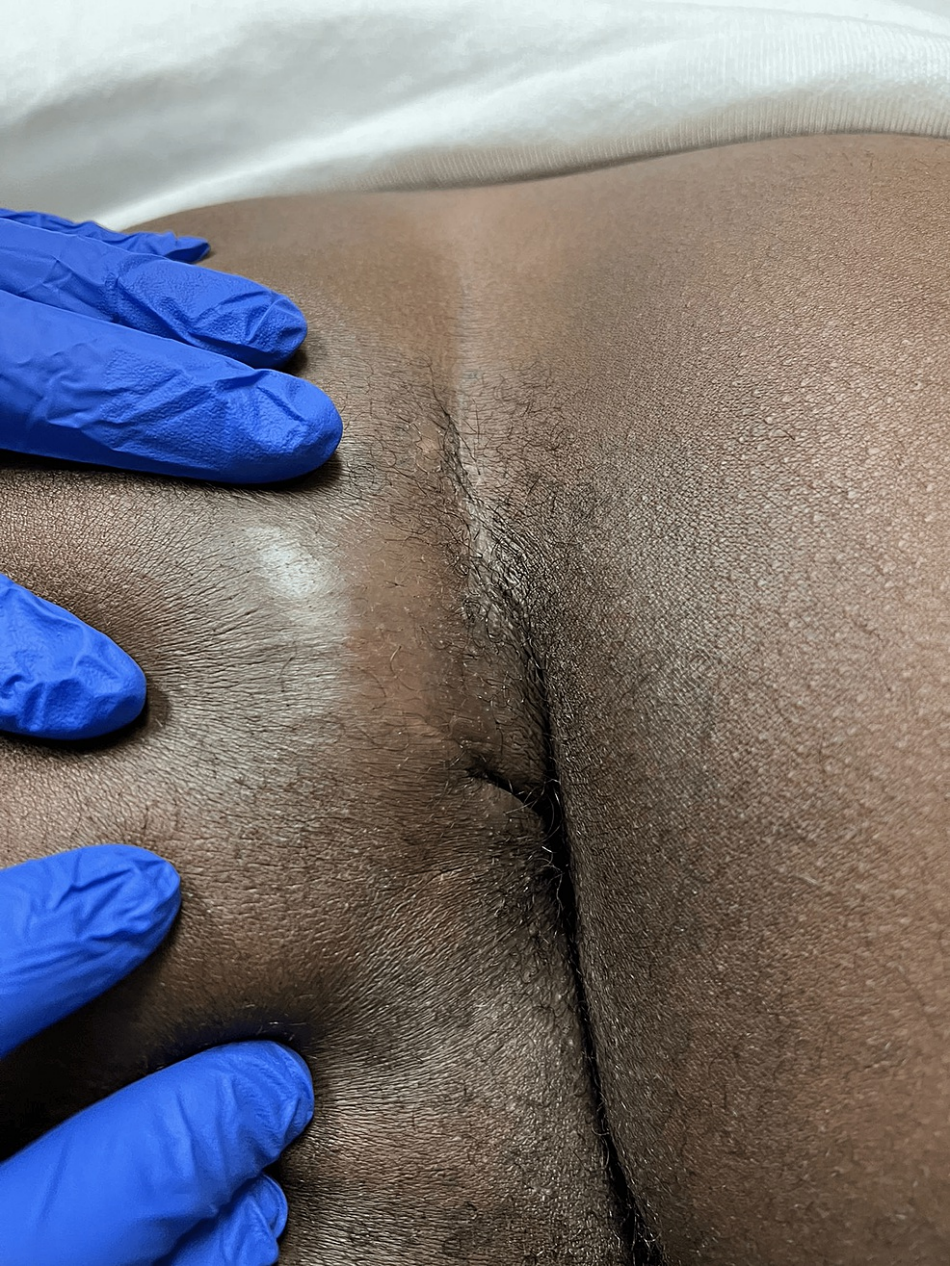
Gross view of left perianal abscess upon admission to hospital

The patient presented in this case report is a 30-year-old African American male with a decade-long past medical history of HS leading to recurrent abscess formation and subsequent infections requiring incision and drainage procedures. The patient was previously prescribed Humira by a dermatologist due to flare-ups; however, due to lack of insurance coverage, the patient did not start this medication and is currently not taking any type of prophylactic treatment. The patient smoked tobacco daily and weighed 100 kg at the time of initial admission. The patient first presented to the emergency department with complaints of severe pain for approximately five days in his perianal area on the left side which has prevented him from lying or sitting down. On physical examination, it was determined that the patient had a left perianal abscess requiring incision and drainage which was subsequently performed, and the patient was discharged the same day. Following this procedure, the patient continued to have recurrent emergency room visits every few months for the same problem, again, requiring incision and drainage, with wound packing and closure. Each time the patient was discharged from the hospital, he was sent home with 600 mg of Ibuprofen every eight hours (q8h) for pain, 15 mg ketorolac intramuscular injection once, and 300 mg clindamycin q8h for 10 days to prevent infection. The wound care department was also consulted to help with educating the patient on his condition and how to best manage the affected area at home with suggestions such as sitz baths and proper frequent hygiene. Months of recurrent hospital visits eventually took a toll on the patient's mental health, as he presented to the emergency department with suicidal ideations. He expressed hearing voices telling him he is worthless and that he would be better off dead, which he attributed to suffering from depression due to his chronic medical condition. Given the patient's past medical history, which includes major depressive disorder (MDD) and generalized anxiety disorder (GAD), and his current situation of not being able to access psychiatric medications due to a loss of insurance coverage, the emergency room physician strongly recommended hospitalization for the patient's safety and stabilization. Three months later, the patient presented back to the emergency department due to a perianal abscess on his left buttock for the past four days, preventing him from being able to lay on his back or sit; however, he denied pain with defecation (Figure 1).

**Figure 2:**
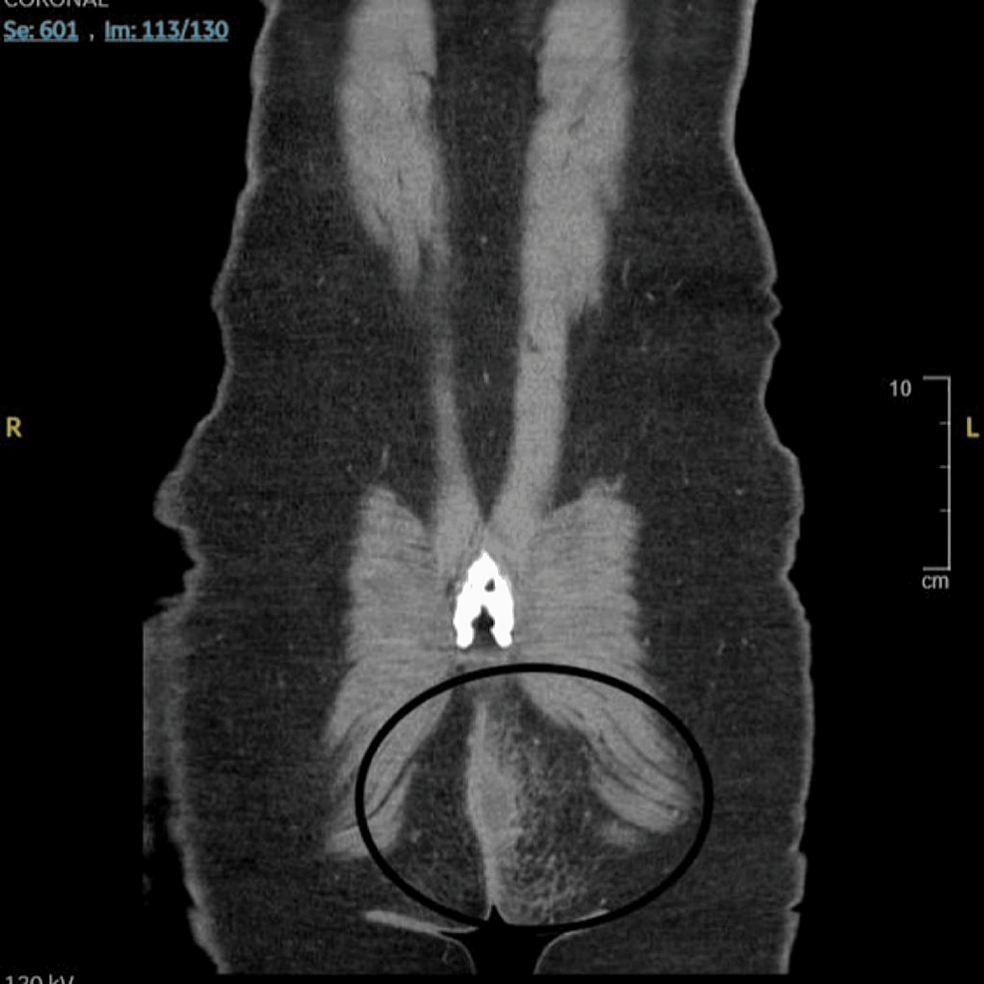
Coronal cross-sectional CT showing left perianal abscess

**Figure 3:**
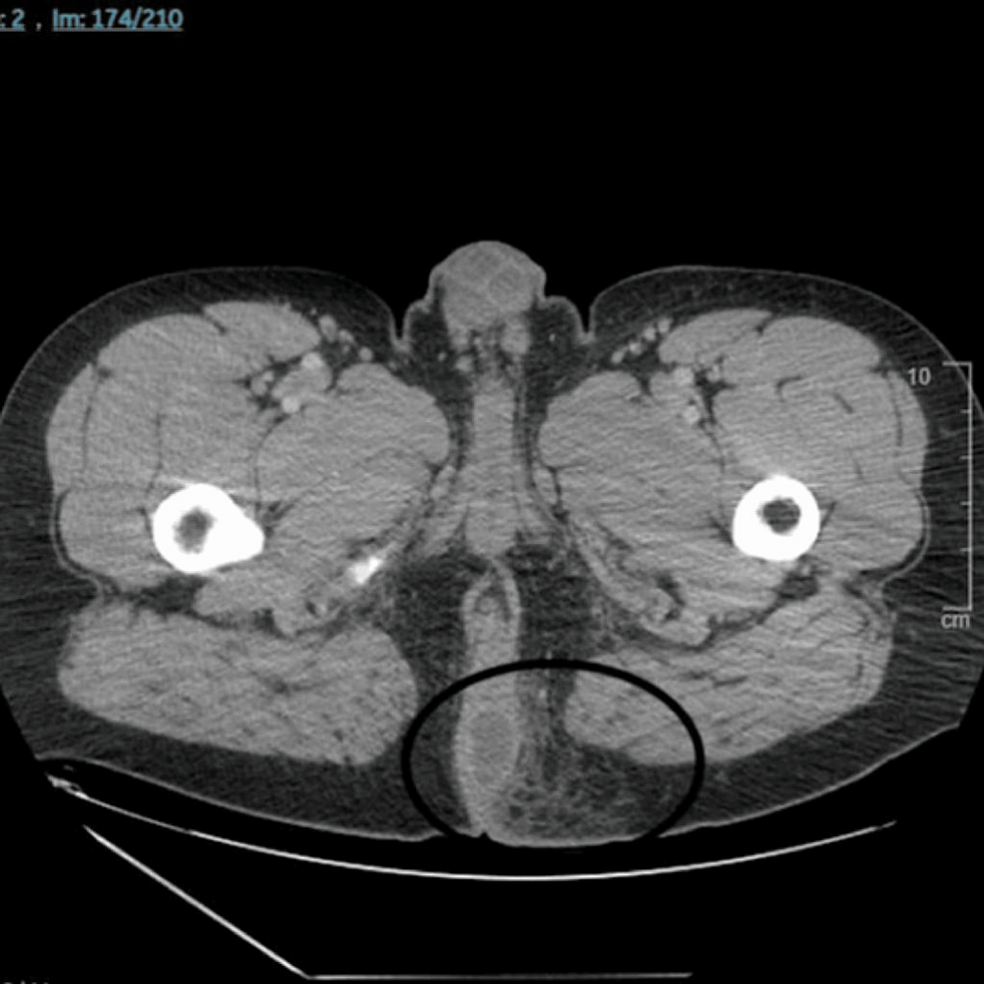
CT abdomen and pelvis w/ IV contrast showing left perianal abscess in the subperitoneal space measuring 3.4 cm x 1.9 cm (black circle), with stranding in the adjacent soft tissues

The patient had no signs of systemic infection such as fever or chills but had a blood pressure value of 202/85. A CT scan of the abdomen and pelvis was performed due to complaints of abdominal pain, with results displaying a 3.4 x 1.9 cm left perianal fluid collection with fat stranding in the adjacent soft tissues (Figures 2, 3).

**Figure 4:**
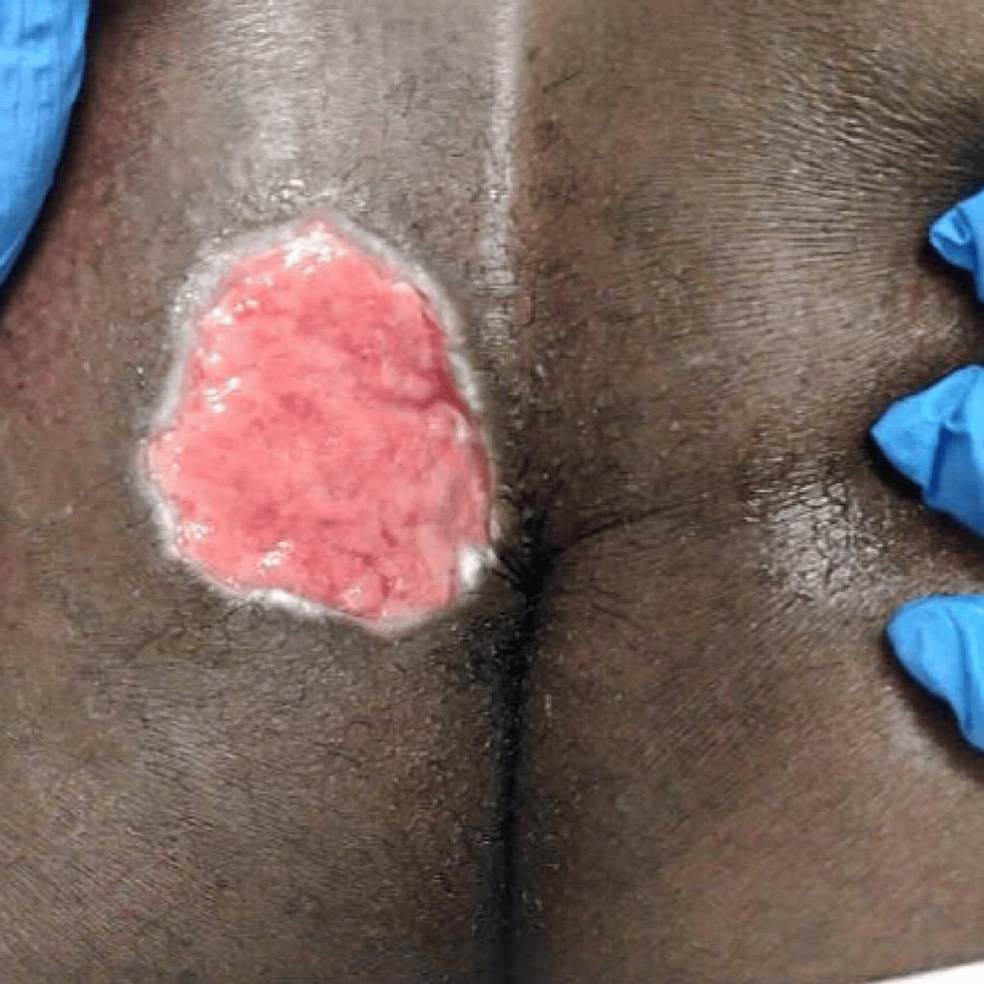
POD 2 following wound debridement POD: post-operative day.

The same day, the patient was taken into the operating room where a complex debridement and incision and drainage were performed. The debrided tissue from the perianal abscess measured 5.3 cm x 4.2 cm and was excised to a depth of 1.5 cm. The surgical pathology report of the specimen displayed ulcerated and necrotic skin, as well as underlying fibroadipose tissue that was accompanied by an abscess with foul-smelling pus and acute hemorrhage. Cultures were collected which demonstrated no anaerobic bacterial growth. The patient was treated post-operatively with 2 g of IV ceftriaxone once daily, 500 mg of IV metronidazole q8h, 0.5 mg of IV hydromorphone as needed (PRN), and 4 mg of IV ondansetron PRN. Post-operative day (POD) 2, a wound vacuum-assisted closure (VAC) was placed, and the wound bed appeared beefy red with 90% granulation tissue and 10% slough (Figure 4).

Moderate discharge was observed but no odor, eschar, or signs/symptoms of infection. Wet-to-dry dressings were placed on the wound, and the infectious disease department was consulted for an opinion on treatment moving forward to decrease the recurrence rate of this disease, in which they suggested checking the patient's G6PD levels as benefit from immunomodulation with dapsone may prove beneficial. The patient's G6PD blood level value was reported to be 213 indicating a low normal value (the normal reference range for males aged 18-30 years old is between 156 and 397 units/trillion red blood cells (RBCs)) and was ultimately excluded as a possible treatment option. On POD 4, IV ceftriaxone and metronidazole were discontinued, and the patient was switched to an outpatient regimen of 500 mg metronidazole tablet q8h and a 100 mg doxycycline capsule twice daily and was ultimately discharged from the hospital. Prior to discharge, initiation of Infliximab, also known as Remicade, a TNF-alpha inhibitor medication, was encouraged by the Department of Infectious Disease in order to decrease the number of future flare-ups of HS. Instructions to return once per week for continued wound care and wet-to-dry dressing changes were also given. These weekly wound care visits over the next one month showed good healing of the surgical site with increased growth of granulation tissue.

## Discussion

This case report is noteworthy due to HS affecting a large proportion of patients of low socioeconomic status, women, and African American individuals. This illness tends to recur and can be the cause of repeated visits seeking medical care. It is also important to focus on diagnosing this condition allowing for prompt treatment initiation since this illness can be commonly misdiagnosed as ingrown hairs or other infections, which ultimately prolongs recovery. Due to this fact, it is important to understand the disease’s pathophysiology for a more robust understanding of this disease. HS is a chronic disease characterized by recurrent, painful, deep-seated, rounded nodules and abscesses of apocrine gland-bearing skin. It is most commonly said to be due to clogging of hair follicles with sweat and keratin from the skin, subsequently leading to the proliferation of bacteria and an inflammatory response due to follicle rupture [6]. The onset of HS most commonly occurs post-puberty, with the most common age of onset range occurring in the third decade of life. Due to the chronic nature of HS, it may progress to subcutaneous extension leading to indurations of the sinus tracts, also known as "tunneling", having a vast effect on one’s quality of life. Dermal tunnels in HS are unique structures made up of stratified squamous epithelium that recapitulate the structure of the overlying epidermis and produce active inflammatory mediators. This is in contrast to other tunnel-like structures that are found in other chronic inflammatory conditions such as fistula formation in Crohn’s disease, which do not recapitulate mucosal structures with the same degree of fidelity. The mechanisms behind tunnel development are unclear; however, it is hypothesized that these tunnels derive from the aberrant keratinocyte outgrowth from the outer root sheath of the follicle [7]. The Hurley staging system is a classification used to determine the severity of disease in patients with HS. This system divides care among three major stages that guide the surgical team on the correct approach. However, this system does not consider the inflammatory component of the disease. During Hurley stage I, there can be a single abscess formation without any sinus tract scarring. In Hurley stage II, the patient presents with simple or multiple separated recurrent abscesses with the presence of sinus tracts and scarring. Lastly, at Hurley stage III, there are multiple continuous and connected sinus tracts that are spread throughout the entire region [8]. Our patient was classified as Hurley scales I and II which aided us on the surgical approach part of the treatment.

With respect to the location of HS, both the axillary and inguinal glands involvement are most common in females, whereas perianal and buttock localization is more prevalent in males, as seen in this patient. A retrospective study of 43 patients with perianal HS was conducted, in which 40 patients (93%) were male and three (7%) were female. The median age at presentation of HS in this group was 29 years. In the case of perianal HS, such a location of disease can be misdiagnosed since it develops adjacent to the anal canal and other soft tissues, commonly mistaken for perianal fistulizing Crohn’s disease (PFCD). PFCD can be differentiated from HS only by clinical features and pathological manifestations as both HS and PFCD have abscesses or sinus tracts with perianal pain, redness, itching, bleeding, and increased purulent secretions [9]. Treatment options for HS are varied and are based on the stage of disease presentation, as early nodular lesions may be treated with antibiotics for the acute stage and long-term antibiotics and zinc salts may be useful as a maintenance treatment. TNF-alpha inhibitor medications have been used in rare severe cases. Surgical treatment options include incision with or without drainage for limited abscesses; limited excisions and healing with secondary intention or flaps and grafts have been found to be the only curative procedure in cases of severe advanced disease. In addition, it is crucial to note that the surgical-wide incision approach used as a treatment option in severe cases of HS was more successful in preventing the recurrence of the perianal type of this illness in comparison with other surgical approaches [10]. A meta-analysis of 715 identified studies showed a lower recurrence rate of wide excision surgical technique to be 8% and a higher recurrence rate of partial/local excision surgical technique to be 34%. Our patient was surgically managed using the wide excision surgical technique which has widely been proven to have a lower recurrence rate of perianal HS [10].

In addition, dapsone has been recently considered as a possible treatment option for HS, as it was in our patient, due to its antimicrobial, bacteriostatic, and also anti-inflammatory properties. Dapsone’s main mechanism of action is to inhibit the synthesis of dihydrofolic acid by blocking the bacterial enzyme dihydropteroate synthase. Seven studies were conducted, involving a combined patient cohort of 135 individuals. Among these patients, 62.2% exhibited varying degrees of improvement post-treatment. However, it is worth noting that only three of the seven studies exclusively employed dapsone monotherapy. This makes it challenging to definitively attribute the observed benefits solely to dapsone therapy, as some of the therapeutic effects might have been contributed by other treatments [5]. However, a systematic review of the use of dapsone as a treatment modality in HS in 2022 found that dapsone in combination with other medications had either a slightly or clinically significant improved health status following treatment. Current guidelines indicate that dapsone can be used in the treatment of mild to moderate HS (i.e., Hurley stages I and II); however, the findings from this systematic review show that dapsone can also be effective in the treatment of severe HS (i.e., Hurley stage III). The use of dapsone in HS was found to have dual functionality with both anti-inflammatory properties and antimicrobial properties, compared to only antimicrobial treatments alone (tetracyclines, clindamycin, metronidazole) [11]. The use of dapsone helped to lower anti-inflammatory markers such as C-reactive protein (CRP) levels in the patient population of this study which may be investigated further to determine if dapsone can be used as a maintenance treatment modality for HS. The most recommended antimicrobial agents for the treatment of HS include macrolide antibiotics such as clindamycin, tetracyclines, rifampicin, metronidazole, and moxifloxacin. Unfortunately, there have been an increasing number of reports of bacterial resistance against these agents, making dapsone a promising treatment option [5].

Lastly, due to the chronic and often disfiguring nature of its physical ailments including pain and increased unpleasant odor due to purulent discharge, HS can have a tremendous burden on mental health. It has been reported that 33% of patients with HS suffer from major depressive disorder (MDD), and 12% from generalized anxiety disorder (GAD) within their lifetime [12]. This patient had a past medical history of psychiatric hospitalization due to MDD, suicide ideation, and GAD, as well as a history of being previously prescribed a regimen of the serotonin reuptake inhibitor (SSRI), sertraline. Despite this past psychiatric history, his mental health needs were not addressed during his most recent visit for the perineal abscess which was subsequently treated surgically.

Furthermore, studies have found that SSRIs can exhibit antimicrobial properties, with sertraline found to be the most potent. Sertraline exhibits intrinsic antibacterial activity against strains of *Staphylococcus aureus*, which has been one of the species of bacteria found in lesion tissue samples from patients with HS [13]. The proposed mechanism explaining the antimicrobial action of SSRIs is the inhibition of bacterial efflux pumps, which may explain the synergistic effect of SSRIs in combination with tetracyclines and fluoroquinolones [14]. Therefore, effectively managing HS patients’ psychiatric needs will not only help improve their quality of life but may also be beneficial in the management of their HS.

## Conclusions

In conclusion, HS is a complicated disease that has many effects on not only one’s physical health but mental health as well. Diagnosing this condition early in patients is key to starting them on the right track to recovery with supportive care options, as well as medicinal treatments. Although the use of dapsone has yet to be an FDA-approved medication for use in HS, medications used for the treatment of HS as a whole are an under-researched area, and more work needs to be done to create and develop a preventative and permanent solution. In addition, barriers to one’s access to medical care, such as lack of insurance coverage, no mode of transportation to healthcare clinics, and potential language barriers between patients and healthcare providers, are all potential limitations to a patient with possible HS receiving a timely diagnosis and initiation of treatment. By improving these factors, patients receive a faster diagnosis and treatment plan, thus decreasing the risk of complications and improving their overall health outcomes.
